# Lipomatous/Extensively Vacuolated Ependymoma with Signet-Ring Cell-Like Appearance: Analysis of a Case with Extensive Literature Review

**DOI:** 10.1155/2017/8617050

**Published:** 2017-02-14

**Authors:** Miguel Fdo. Salazar, Martha Lilia Tena-Suck, Alma Ortiz-Plata, Citlaltepetl Salinas-Lara, Daniel Rembao-Bojórquez

**Affiliations:** ^1^Department of Neuropathology, National Institute of Neurology & Neurosurgery “Manuel Velasco Suárez”, Tlalpan, Mexico City, Mexico; ^2^Laboratory of Experimental Neuropathology, National Institute of Neurology & Neurosurgery “Manuel Velasco Suárez”, Tlalpan, Mexico City, Mexico

## Abstract

“Lipomatous” and “extensively vacuolated” are descriptive captions that have been used to portray a curious subset of ependymomas distinctively bearing cells with a large vacuole pushing the nucleus to the periphery and, thus, simulating a signet-ring cell appearance. Here, we would like to report the first ependymoma of this kind in a Latin American institution. A 16-year-old boy experienced cephalea during three months. Magnetic resonance imaging scans showed a left paraventricular tumour which corresponded to anaplastic ependymoma. Intriguingly, it was also composed of cells with single or multiple hollow cytoplasmic vacuoles sometimes giving a signet-ring cell-like configuration. Immunolabeling of these showed membrane positivity for GFAP, PS100, and CD99, while Ki-67 expression was null. Ultrastructural examination of retrieved paraffin-embedded tissue showed the presence of scarce microlumina filled with microvilli but failed to demonstrate any content in such optically empty vacuoles as only scant granulofibrillary debris was observed. A schism prevails at present regarding these unusual morphological variants, being either “lipomatous” or “vacuolated” based mainly on the EMA immunoprofile. This, however, is a misappropriate approaching. Could it be that perhaps we are dealing with the same histopathological entity or it may simply happen that fixation and artefacts cannot allow for their proper identification?

## 1. Introduction

Ependymomas are neuroepithelial neoplasms with well-recognised and widely known histopathological subtypes such as the tanycytic, papillary, and clear cell variants [1, 2]. Apart from this morphological spectrum, a new genetically defined entity, the recently introduced “RELA fusion-positive” ependymoma, is now included in the 2016 version of the WHO classification of CNS tumours [2]. In spite of this trend, curious examples of ependymomas with cells mimicking an adipocyte-like appearance, and to some extent bearing a cardinal signet-ring cell phenotype, have been described since the midnineties in the neuropathology lore. Accordingly, almost thirty cases with such traits have been reported worldwide [3–15] while we document another case in a nonpreviously concerned region such as Latin America (Table 1).

## 2. Case Report

**Figure 1:**
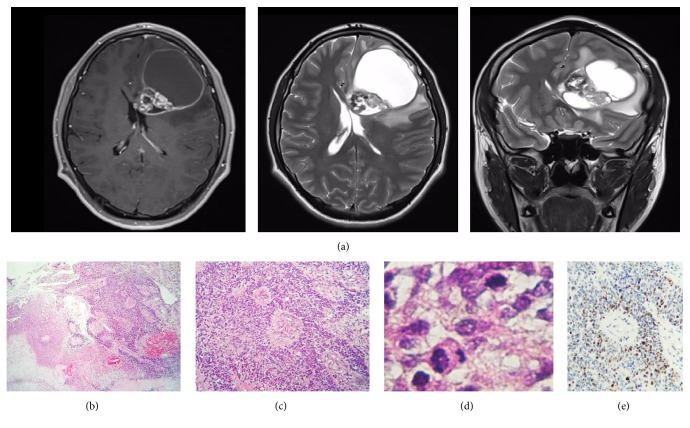
Magnetic resonance imaging scans/histopathological findings (anaplastic component). (a) Postcontrast T_1_ (right), T_2_-weighted (center), and fluid attenuated inversion recovery, FLAIR, sequence (left) in axial and coronal planes demonstrating a left periventricular, partially cystic, and bulky tumour. (b) Panoramical low magnification photomicrograph showing a densely populated neoplasm apparently assembling rosettes (right upper field) and surrounded by geographic necrosis (left lower field). (c) High magnification photomicrograph of the right upper field shown in (b). There are multiple perivascular pseudorosettes denoting conspicuous microvascular proliferation. (d) Mitotic activity in a high-power field. (e) Ki-67 immunolabeling index (~50%).

**Figure 2:**
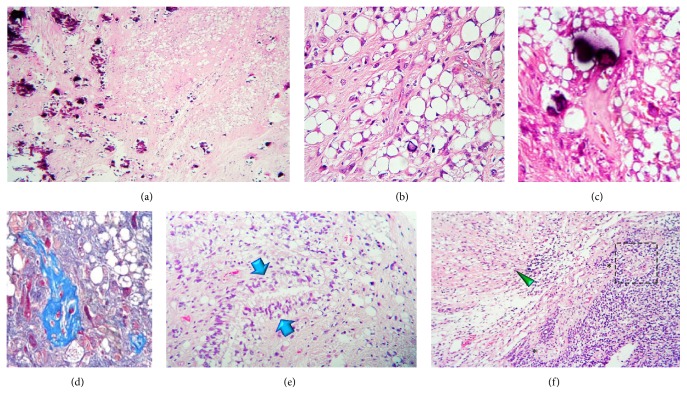
Histopathological findings (lipomatous/vacuolated component). (a) Low magnification photomicrograph showing numerous dystrophic calcification foci (left field) around areas resembling fat lobules (right upper field). (b) Signet-ring cells with an optically empty cytoplasm resembling adipose tissue. (c) Heavily hyalinised vessel with dystrophic calcification in a high-power field. (d) Densely collagenised vessel seen with Masson's trichrome in a high-power field. (e) Fortuitously found ependymal channel (blue arrows). This one attests partial vacuolation of the ependymal lining. (f) Boundary zone between a signet-ring cell area (green arrowhead) and the anaplastic component (right field). The black asterisks plot a long tailed blood vessel which ends in a microvascular proliferated glomerulus-like head.

**Figure 3:**
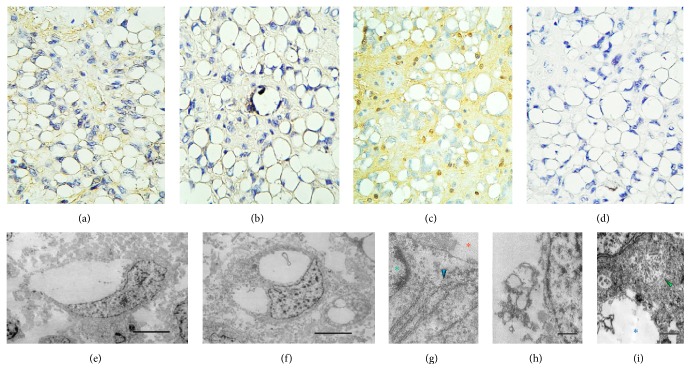
Immunohistochemistry panel/transmission electron microscopy. (a) Glial fibrillary acidic protein (GFAP). (b) CD99. (c) PS100. (d) Ki-67 (MIB-1). (e, f) Uni- and multivacuolated cells featuring void lumina. (g) Rough endoplasmic reticulum (blue arrowhead) near an empty vacuole (orange asterisk). There is no apparent connection between them. On the left side, a small part of the cell's nucleus can be seen (green asterisk). (h) Hollow intracytoplasmic lumen containing granulofibrillary debris, slightly resembling degenerated microvilli or organelles, next to the nucleus (right field). (i) Microrosette filled with microvilli (green arrowhead) lying close to a dilated vacuole with granulofibrillary material (blue asterisk).

A 16-year-old boy without any pathological, genetic, or familial history of clinical relevance suffered from headaches during May 2016. A few weeks later he began to experience intermittent diplopia as well as left eye esotropia, thus attending to a primary care physician who sent him to our institution. Magnetic resonance imaging (MRI) scans revealed a left intra- and paraventricular frontal tumour with cystic parts and manifestly enhancing edges after contrast medium administration (Figure 1(a)). A hard calcified lesion with a yellow-hued rim was excised by the surgeon. Histopathological examination of it disclosed a densely populated neuroepithelial neoplasm formed by sheets of small, mitotically active, hyperchromatic glial cells lumping around vessels exhibiting microvascular proliferation (Figures 1(b)–1(e)). The immediate impression was anaplastic ependymoma. Nevertheless, to our surprise, there were also some other regions displaying an entirely different morphology, and which composed approximately 50% of the sample, featuring cells with a large optically clear vacuole pushing the nucleus to the periphery resembling either mature adipose tissue or signet-ring cells (Figures 2(a) and 2(b)). Periodic acid-Schiff (PAS), alcian blue, and mucicarmine stains failed to demonstrate any content in such vacuoles. Blood vessels in these areas did not show microvascular proliferation but instead were hyalinised and calcified (Figures 2(c) and 2(d)). The ependymal nature of these curious neoplastic cells was also not evident; however, we incidentally came across some canalicular formations lined by ciliated cylindrical cells exhibiting the same vacuolative changes as the signet-ring-like ones (Figure 2(e)). Furthermore, there were transition zones between the lipomatous/vacuolated and the anaplastic component (Figure 2(f)). The immunohistochemical reactions performed showed a faint membranous staining for glial fibrillary acidic protein (GFAP), CD99, and PS100 (Figures 3(a)–3(c)) while no reaction towards epithelial membrane antigen (EMA) was observed. Conversely to the anaplastic part of the neoplasm, the Ki-67 proliferation index was extremely low here (<1%) (Figure 3(d)). Paraffin-embedded tissue was retrieved for transmission electron microscopy which, again, failed to recognise any particular substance inside the vacuoles, as just void gaps were persistently observed (Figures 3(e) and 3(f)). No relationship with endoplasmic reticulum or other organelles could be identified (Figure 3(g)). Intriguingly, granulofibrillary material was eventually found attached to the vacuoles' circumference barely resembling some kind of degenerated component (Figures 3(h) and 3(i)). Small intracytoplasmic lumina filled with multiple microvilli* (microrosettes)* were also recognised, confirming ultrastructurally the ependymal nature of the vacuolated cells (Figure 3(i)).

Hence, since no distinctive content was unequivocally and satisfactorily characterised in postfixated tissue, this case was regarded as a* lipomatous/extensively vacuolated ependymoma with signet-ring cell-like appearance *plus an accompanying anaplastic component.

## 3. Discussion

Lipomatous/vacuolative change with signet-ring cells is a very uncommon phenomenon seldom seen in ependymal neoplasms. For instance, Sharma et al. [3] retrieved just 5 examples from 193 ependymomas assessed during a period of 19 years, which roughly represents 2.59%. This percentage might not seem low enough but when considering that the 193 ependymal tumours comprised 2.1% of all their cases, then the former calculated ratio dramatically falls to 0.054%. Likewise, Gessi et al. [4] collected 6 cases from 1994 to 2010 and determined an overall incidence of 0.23%.

The study of deceivingly hollow intracytoplasmic lumina (ICL) in ependymomas is not a new subject: in 1993, a beautiful paper from the master hand of Ho et al. [16] rigorously and elegantly described, by means of transmission electron microscopy, the different evolving stages in the morphogenesis of normal appearing ICL. Two different types of them were characterised: ciliated and nonciliated ICL; the first subset arose from distension of periciliary cisterns with progressive enlargement and ensuing opening into the extracellular or intercellular space. They were packed with numerous microvilli as well as cilia often displaying microtubular abnormalities. Conversely, the second group of nonciliated ICL were more arcane in origin, as they contained only scarce microvilli and loose or condensed granulofibrillary material. Also, they were not related to Golgi apparatus, endoplasmic reticulum, or mitochondria and their traffic across the cytoplasm was more inert. Hence, they hypothesised that these could represent a degenerative form of ciliated ICL or result from invagination of the extracellular space. In the following year, Mierau et al. [5] wrote about two brain tumour cases bearing obvious ependymal traits but also displaying the unusual presence of scattered cells with a single large vacuole compressing the nucleus into a crescent at one edge of the cytoplasm, shaped, thus, in the classical morphology of signet-ring cells. Ultrastructural examination of these unearthed the presence of attenuated microvilli and occasional cilia, very similar to the ciliated ICL formerly detailed by Ho et al. [16]. Three years later, Hirato et al. [6] reported a new case of ependymoma with vacuolar features and signet-ring cells which were not stained with PAS, alcian blue, or oil red O. The electron microscopy assessment described variably enlarged ICL without microvilli but with some granulofibrillary debris inside, akin to the nonciliated ICL of Ho et al. [16]. They suggested this material could represent degenerated organelles and that the vacuoles might arise from deteriorated endoplasmic reticulum, as some of the ICL showed attachment of ribosomes. During the ensuing period, Ruchoux et al. [7] analysed other three ependymoma cases exhibiting signet-ring cell-like features; however, since MRI scans supposedly hinted to the presence of lipid in one of such tumours, they were able to demonstrate it by means of fat-soluble dyes in fresh tissue obtained from this single case. Likewise, Takahashi et al. [8] independently portrayed a “lipidized” variant of ependymoma confirming the presence of variable sized osmiophilic lipid droplets in the material submitted for ultrastructural examination.

Thus, given the aforementioned background, inquiries such as the following become appropriate: ① how should we cope with all those cases where vacuolative features are unmistakably seen but where formaldehyde postfixation and paraffin embedding cannot reliably rule out a lipid removal effect from the sample assessed? ② Does a consistently ancillary test exist to discriminate one from the other (extensively vacuolated versus lipomatous)? Many authors have used EMA immunostaining as a key diagnostic tool for this last purpose, claiming that its absence in the signet-ring-like cells is supposedly observed in the lipid-storing variant [3, 4, 7, 9]. This, however, represents a questionable protocol due to the high variability of EMA immunolabeling among ependymomas [1], as well as misinterpreted data regarding Hirato's results [6]. Indeed, he stated that almost all tumour cells in his case were negative for EMA, yet, with some scattered granular or microvesicular positive structures, which is exactly the same pattern described in some cases phrased as lipomatous [7, 8]. Conversely, perimembranous immunolabeling has also been demonstrated in both [4, 10]. Thus, given this ambiguity, we consider that demonstration of a stored material with properties akin to neutral lipids, either by means of histochemistry or by transmission electron microscopy, is the only guaranteed method to prove the fat-bearing nature of such neoplasms. Interestingly, by applying this sole criterion, only three authentically proven cases of lipomatous ependymomas exist and are recorded [7, 8, 10] (Table 1). This, however, is an utterly impossible task in postfixated tissue; even by means of electron transmission microscopy only hollow spaces will be found. On the other hand, some other authors have claimed its early suspicion in MRI scans [7, 10, 11] which, however, possess their own limitations mainly related to the amount of fat in the analysed tissue and in the type of fat suppression technique employed [17]. We might suspect as well cases where xanthochromic fluid was found intraoperatively, like in the examples of Ruchoux et al. [7], Chang and Finn [11], Ertan et al. [9], or in ours, nevertheless, this is a quite subjective and nonspecific finding.

As far as we know, lipidization amidst central nervous system neoplasms is an intriguing and poorly understood phenomenon which lacks a convincing explanation. Though initially thought to be some kind of metaplasia [7], this seems unlikely in the light of the preserved immunohistochemical and ultrastructural basic traits of the altered cells. Again, new questions turn fundamental: ③ do the ependymoma subtypes analysed herein constitute a fat-storage metabolic cell imbalance and a degenerative vacuolation process, respectively? ④ Are both changes mutually exclusive phenomena? ⑤ How important is it to properly identify and diagnose each one? It has been stated that intracytoplasmic vacuolation of ependymal cells can be experimentally observed in the hypoxic state [6]; interestingly, we noticed the presence of thick hyalinised vessels in the same fields as the signet-ring-like cells (Figures 2(c) and 2(d)), a finding that has also been described by some other authors [5, 12]. This possibly points towards a true degenerative event that can be rarely but potentially seen in any kind of ependymoma. Moreover, if this is real, it is then possible to advocate a feasible pathway to link both variants, lipomatous and extensively vacuolated, like this: hypoxia → organelle vacuolation (ribosomes, rough endoplasmic reticulum) → protein synthesis impairment → defective lipid transportation with storing of fat droplets. Likewise, the following could also be correct: organelle vacuolation → disaggregation of structural membrane lipids → accumulation of glycerophospholipids in the cell's cytoplasm. Nevertheless, from a clinical point of view, this long scholar discussion might be utterly sterile as none of them appears to be relevant on neither prognosis nor treatment. It had been speculated that ependymomas bearing these signet-ring-like traits behave in a similar way as conventional ones [3], an idea that has been endorsed by the low proliferation indexes frequently reported in them (Table 1). Likewise, aggressive and recurrent examples have been described, though they usually come in association with an anaplastic or a clear cell ependymoma component [3, 6, 11, 12] (Table 1).

There are two more cases reported as* signet-ring cell ependymomas* in the worldwide literature shrewdly described by the pen of both Cenacchi et al. [18] and Mizuno et al. [19]. They, however, do not correspond to the lipomatous or vacuolated patterns discussed here as they are chiefly characterised by secretion of alcian blue positive material, thus, in fact, representing true* mucin-secreting signet-ring cell ependymomas*. Likewise, Jouvet et al. [20] wrote about a lipomatous neoplasm with ependymal differentiation yet bearing a chief neurocytomatous morphophenotypes and immunophenotypes.

The main differential diagnoses to ponder in a brain tumour case with prominent signet-ring-like cells resembling adipocytes are metastasic signet-ring cell adenocarcinoma, particularly from breast or gastrointestinal tract, intracranial lipomatous hamartoma, and liponeurocytoma [5, 10, 20]. Fortunately, an appropriate immunohistochemical panel can easily discard them by testing the immunoreactivity towards cytokeratins (AE_1_/AE_3_ cocktail, CK7, and CK20), GFAP, and neuronal markers (NeuN, synaptophysin, and neurofilaments). Another potential pitfall in our setting is clear cell ependymoma; indeed, some of the reported lipomatous/extensively vacuolated signet-ring cell ependymomas coexist with this variant [6, 12, 13] (Table 1). Nonetheless, they are morphophenotypically different as the former displays an oligodendroglia-like appearance with perinuclear clear halos due to glycogen buildup [1].

In conclusion,* lipomatous *and* extensively vacuolated *are unconventional histopathological patterns seen in ependymomas probably as common degenerative phenomenon secondary to a hypoxic insult and which, once detected in postfixated tissue, are very difficult to accurately discriminate from one another. Despite this distinction is not decisive for clinical purposes, its confusion with more common lesions such as metastasic signet-ring cell adenocarcinoma is detrimental to the patient's treatment and management. It is also important to keep recording them in order to achieve a better understanding of both their biology and histopathogenesis; hence, we add a new case to the global registry as well as the first one known to Latin American population.

## Figures and Tables

**Table 1:** Lipomatous and extensively vacuolated ependymomas with signet-ring cells case list.

Case #.	Year	Author (country)	Age/gender	Location	Phrased diagnosis	Additional histopathological features (VÇ areas)	EMA (VÇ)	PI (VÇ)	Ultrastructural findings (VÇ)	Associated neuroepithelial component	Follow-up
1	1994	Mierau et al. [5] (U.S.A.)	12♀	Left parietooccipital region	Signet-ring cell	Focal calcification	NR	NR	Large vacuoles with attenuated microvilli	Conventional ependymoma	NS
2			44♀	4th ventricle		Thick collagenous collars around blood vessels	NR	NR	Numerous large vacuoles lined by microvilli and cilia	NS	NS

3	1997	Hirato et al. [6] (Japan)	2♀	Left occipital lobe	Extensively vacuolated	PAS (−) alcian blue (−) oil red O (−)	Negative (some granular or microvesicular immunopositive structures in cytoplasm or intercellular spaces)	<1%	Numerous vacuoles of different size without microvilli, some of them fused with each other, with attached ribosomes or containing granulofibrillary material simulating degenerated mitochondria	Clear cell ependymoma	Recurrence after 1 y

4	1998	Takahashi et al. [8] (Japan)	49♀	Spinal cord (T12-L1)	Lipidized (foamy)	Densely collagenised stroma sudan III (+)	Positive (granular or microvesicular structures in cytoplasm and intercellular spaces)	NR	Osmiophilic fat droplets of variable size as well as large vacuoles with scarce microvilli	Conventional ependymoma	No recurrence after 6 m

5	1998	Ruchoux et al. [7] (France)	13♂	Left frontal lobe	Lipomatous	Lymphocytic clusters oil red O (+)	Negative	<1%	Osmiophilic fat droplets with smooth margins as well as cystic vacuoles with scarce microvilli	Conventional ependymoma	No recurrence after 2 y
6			16♂	Left parietooccipital region		Few calcium foci mucin stains (−)	NR	<1%	No *e*^−^*μ*	Anaplastic ependymoma	NS
7			42♀	3rd ventricle		None	NR	NR	No *e*^−^*μ*	Papillary ependymoma	NS

8	1999	Vajtai et al. [12] (Hungry)	64♂	Left cerebellar hemisphere	Signet-ring cell	Labyrinthine-hyalinised vessels PAS (−)	Positive (membranous and dot-like patterns)	<1%	^*∗*^Vacuoles with scant degenerated microvilli, distended rER, coupling of tumour cells around dilated intercellular lumina	Clear cell ependymoma	Two consecutive surgeries at 8 m interval

9	2000	Sharma et al. [3] (India)	18♂	Left parietal lobe	Lipomatous	None	Negative	<20%	No *e*^−^*μ*	Anaplastic ependymoma	Recurrence after 2 y
10			17♂	Posterior fossa		None	Negative	<20%	No *e*^−^*μ*	Anaplastic ependymoma	Recurrence after 2 y
11			4♂	Right frontal lobe (lateral ventricle-3rd ventricle)		None	Negative	NR	No *e*^−^*μ*	Cellular ependymoma	Recurrence after 4 y
12			35♂	4th ventricle		None	Negative	<1%	No *e*^−^*μ*	Conventional ependymoma	No recurrence after 1 y
13			45♂	Spinal cord (T2-T3)		None	Negative	<1%	No *e*^−^*μ*	Conventional ependymoma	No recurrence after 3 y

14	2001	Chang and Finn [11] (U.S.A)	5♂	Left parietooccipital region (lateral ventricle)	Lipomatous	Patchy calcification	Negative	NR	No *e*^−^*μ*	Anaplastic ependymoma	Two consecutive surgeries at 8 m interval

15	2001	Otani et al. [14] (Japan)	37♀	Spinal cord	Signet-ring cell	NA	NA	NA	NA	NA	NA
16			52♀	Spinal cord		NA	NA	NA	NA	NA	NA

17	2003	Kim et al. [13] (Japan)	59♂	Spinal cord (T3-T4)	Lipidized (foamy)	None	Positive (microvesicular and granular patterns)	<1%	No *e*^−^*μ*	Clear cell ependymoma	NS

18	2005	Onaya et al. [15] (France)	51♂	Spinal cord (T6-T7)	Lipomatous	NA	NA	NA	NA	NA	NA

19	2010	Ertan et al. [9] (Turkey)	35♀	4th ventricle	Signet-ring cell	Lipofuscin, melanin, and rosenthal fibers alcian blue (−)	Positive (dot-like and small vesicular patterns)	<1%	No *e*^−^*μ*	Conventional ependymoma	NS

20	2011	Gessi et al. [4] (Germany)	40♂	Cervical	Extensively vacuolated	PAS-alcian blue (−)	Positive (dot-like and small vesicular patterns)	NR	No *e*^−^*μ*	Conventional ependymoma	NS
21			54♂	Spinal cord (C5-C6)		PAS-alcian blue (−)	Positive (dot-like and small vesicular patterns)	NR	No *e*^−^*μ*	Conventional ependymoma	NS
22			59♂	Spinal cord (C4-C5)		PAS-alcian blue (−)	Positive (dot-like and small vesicular patterns)	NR	No *e*^−^*μ*	Conventional ependymoma	NS
23			30♀	4th ventricle		None	Positive (dot-like and small vesicular patterns)	NR	No *e*^−^*μ*	Conventional ependymoma	NS
24			15♂	4th ventricle		PAS-alcian blue (−)	NR	NR	No *e*^−^*μ*	Conventional ependymoma	Recurrence after 2 y
25			54♀	3rd ventricle-4th ventricle		PAS-alcian blue (−)	Negative	NR	No *e*^−^*μ*	Conventional ependymoma	NS

26	2016	Gaur et al. [10] (India)	13♀	Right lateral ventricle	Lipomatous	PAS (−) alcian blue (−) oil red O (+)	Positive (membranous pattern in some cells)	<3%	Osmiophilic fat droplets	Cellular ependymoma	Recurrence after 1 y

27	2016	Present Case (Mexico)	16♂	Left frontal lobe	Lipomatous | signet-ring cell	Hyalinised vessels with conspicuous dystrophic calcification PAS (−) alcian blue (−)	Negative	<1%	^*∗*^Partially membrane-covered vacuoles of different size without microvilli or cilia but containing granulofibrillary material and not apparently related with rER.	Anaplastic ependymoma	No recurrence after 2 m

*Statistical summary*											
Adults: 17 (62.96%), paediatric: 10 (37.03%), male: 16 (59.26%), and female 11 (40.74%)											
Mean age: 32.63 y | Age range: 2 y to 64 y											
Supratentorial: 11 (40.74%); lateral ventricles: 3, 3rd ventricle: 3, cerebral lobes: 6, infratentorial: 8 (29.63%); 4th ventricle: 6, cerebelar hemispheres: 1 and Spinal Chord: 9 (33.3%)											
Cases with relapsing tumours: 8, relapsed & associated with anaplastic ependymoma: 3, relapsed & associated with clear cell ependymoma: 2											

♂: male, ♀: female, y: years, mo: months, PI: proliferation index (MIB-1 and Ki-67), NR: not requested, NS: not specified, NA: nonavailable data,VÇ: vacuolated cells, *e*^−^*μ*: transmission electron microscopy, rER: rough endoplasmic reticulum, *∗*: material recovered from paraffin-embedded tissue.
